# Supplementary material for: Identification of photoperiod-induced specific miRNAs in the adrenal glands of Sunite sheep (Ovis aries)
Source: Front Vet Sci. 2022 Jul 22;9:888207. doi: 10.3389/fvets.2022.888207 (PMC9354845; doi:10.3389/fvets.2022.888207)
Supplement: Supplementary file 1 [file Data_Sheet_1.ZIP › Supplementary file/miRDeep2 file.zip!/miRDeep2 file/Novel_Structure/SPLP42A2/Novel_169.pdf]

Provisional ID	:	NC_019459.2_6645
Score total	:	0.5
Score for star read(s)	:	-1.3
Score for read counts	:	-1.3
Score for mfe	:	1.6
Score for randfold	:	1.6
Score for cons. seed	:	
Total read count	:	9
Mature read count	:	8
Loop read count	:	1
Star read count	:	0

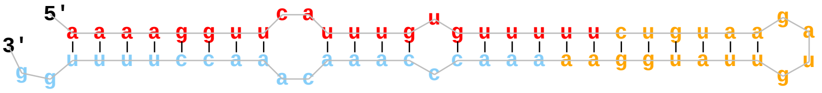

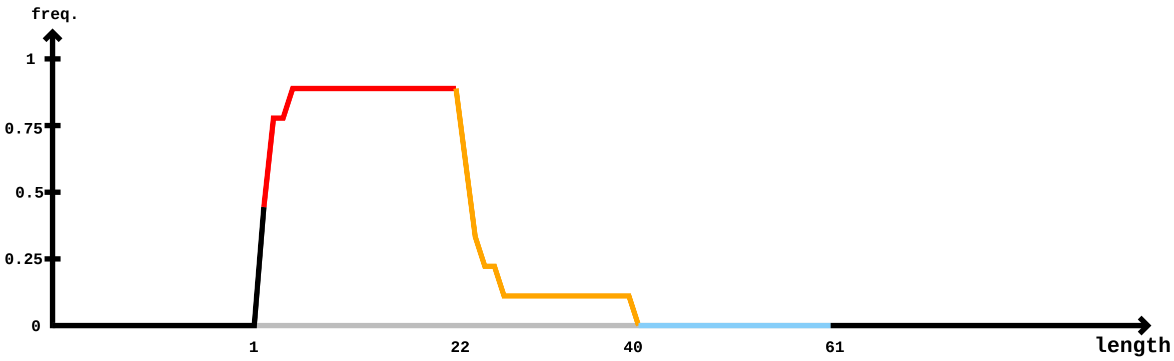

Mature **Star**

```
5'- guggaaauacuagauuggccaaaagguucauuuguguuuuucuguaagauguuauggaaaaacccaaacaaaccuuuuggccaacccaauauaaaacuacucuguauaaguc   -3'    exp
    ......((((.(((((((((((((((((..((((.((((((((((((....)))))))))))).))))..))))))))))))))................)))))))..... reads   mm   sample
    ....................aaaagguucauuuguguuuuu.......................................................................  2     0    seq
    ....................aaaagguucauuuguguuuuuc......................................................................  2     0    seq
    .....................aaagguucauuuguguuuuu.......................................................................  2     0    seq
    .....................aaagguucauuugAguuuuucu.....................................................................  1     1    seq
    .......................agguucauuuguguuuuucugu...................................................................  1     0    seq
    .........................................cuguaagauguuauggaa.....................................................  1     0    seq
```
